# Regulatory T cells: a promising new therapeutic target in ventricular remodeling after myocardial infarction

**DOI:** 10.3389/fimmu.2025.1514335

**Published:** 2025-04-07

**Authors:** Yiran Qin, Mingxuan Li, Haibo Liu

**Affiliations:** ^1^ Department of Cardiology, Qingpu Hospital Affiliated to Fudan University, Shanghai, China; ^2^ Department of Cardiology, Huadong Hospital, Fudan University, Shanghai, China

**Keywords:** regulatory T cells, myocardial infarction, ventricular remodeling, immune regulation, inflammation

## Abstract

Myocardial infarction (MI) is one of the leading causes of death worldwide. It is triggered by thrombosis or vascular occlusion. After MI, damaged cardiomyocytes are replaced by scar tissue, leading to systolic and diastolic dysfunction, followed by adverse remodeling. Regulatory T cells (Tregs), as major immune cells, play a crucial role in post-MI inflammation and immunomodulation. Tregs improve cardiac remodeling after MI through various mechanisms, including inhibiting inflammatory cell infiltration, inducing anti-inflammatory macrophages, suppressing cell apoptosis, regulating fibroblast function, and promoting angiogenesis. The modulation of Tregs number or function may provide novel methods for improving post-MI remodeling. This review describes the immunoregulatory roles of Tregs, their regulatory mechanisms in post-MI ventricular remodeling, and the prospects and challenges for clinical application. However, the exact molecular mechanisms of Tregs in ventricular remodeling remain to be investigated. Although most of the current studies are at the preclinical stage, they hold great potential for further application in the future.

## 1 Introduction

Myocardial infarction (MI) is a global health problem with a serious economic burden, which has been considered the main cause of cardiovascular morbidity and mortality (1). It is an irreversible consequence of coronary artery ischemia. Patients are prone to heart failure after MI, which directly affects their quality of life and prognosis (2). Ventricular remodeling plays a pivotal role in the pathological process of ventricular dysfunction after infarction. It is clinically characterized by ventricular dilation, with other changes including collagen deposition, scar formation, fibrosis, and hypertrophy (3). A variety of drugs have been used to inhibit left ventricular remodeling, such as angiotensin-converting enzyme inhibitors and beta-blockers (4). However, current therapeutic strategies for preventing adverse clinical outcomes remain limited. Therefore, exploring the pathological mechanisms of post-MI ventricular remodeling and developing more effective prevention and treatment strategies are receiving increasing emphasis.

Regulatory T cells (Tregs) are a major type of immune cells involved in MI (5). In recent years, with the deepening of research in immunology and cardiology, the role of Tregs in ventricular remodeling has gradually garnered attention (6–8). Activation of the inflammatory immune system post-MI affects myocardial damage repair. Excessive inflammation results in an increased infarct size and aggravates cardiac remodeling (9). Tregs are a crucial type of lymphocytes that exhibit anti-inflammatory effects (10). They have immunomodulatory and recovery-promoting roles in atherosclerosis (11), acute coronary syndrome (ACS) (12), and chronic heart failure (13). The current review focuses on the role and mechanisms of Tregs in immune-mediated cardiac remodeling following MI, aiming to provide novel perspectives for the treatment and prognosis of MI.

## 2 Mechanisms of ventricular remodeling after MI

Post-MI ventricular remodeling refers to changes in the morphology, size, and tissue structure of the ventricle following myocardial ischemia and hypoxia caused by acute occlusion of the coronary artery. It is a process in which the heart initiates a series of adaptive responses for self-repair and compensation. Long-term and sustained pathological remodeling contributes to heart failure (4). At the histological level, the pathological changes manifest as cardiomyocyte hypertrophy, apoptosis, myofibroblast proliferation, and interstitial fibrosis in the non-infarcted area, leading to the progressive alteration in left ventricular myofibrillar structure (3, 14). The macroscopic manifestations include thinning of the infarcted myocardium, left ventricular dilation, and compensatory myocardial hypertrophy in the infarct border zone. This leads to an increase in both left ventricular end-diastolic and end-systolic volumes over time. The left ventricle gradually transitions from a normal ellipsoidal shape to a spherical one, with abnormal regional wall motion and decreased left ventricular ejection fraction (LVEF) (15).

The mechanisms underlying ventricular remodeling involve several aspects, including metabolic alterations in cardiomyocytes under ischemic and hypoxic conditions, activation of the neuroendocrine system, and changes in the extracellular matrix (16). In the hypoxic environment caused by MI, normal aerobic oxidation in cardiomyocytes is inhibited, and anaerobic glycolysis serves as the main source of energy. Although this metabolic switch can provide energy in a short period of time, the amount of ATP produced is much less than that from aerobic oxidation. Additionally, the accumulation of metabolites such as lactate may lead to intracellular acidosis and impaired cell function (17). After MI, the body rapidly initiates the activation of the sympathetic nervous system and the renin-angiotensin-aldosterone system. These systems collectively promote the development of ventricular remodeling by regulating extracellular matrix deposition and promoting cardiomyocyte hypertrophy and interstitial fibrosis (18). Changes in collagen, fibronectin, and other components of the extracellular matrix lead to disruption of connections between cardiomyocytes and enlargement of the ventricular cavity. Meanwhile, the imbalance between matrix metalloproteinases (MMPs) and tissue inhibitors of metalloproteinases (TIMPs) further exacerbates ventricular remodeling (2, 16, 19).

Immune response plays a significant role in post-MI healing and remodeling (20). Hypoxia and nutrients deficiency result in the death of cardiomyocytes, thereby triggering inflammation (21). Immune cells participate in all stages of MI progression, where they can both exacerbate the death of cardiomyocytes and promote the regeneration of damaged myocardium. Additionally, they regulate the dynamic and complex inflammatory responses (20, 22). Monocytes and macrophages in the infarct area are activated and polarized at different times. They produce pro-inflammatory or anti-inflammatory cytokines, regulate cardiomyocyte proliferation and apoptosis, and influence cardiac remodeling (23). T lymphocytes and B lymphocytes are recruited to the infarct area to remove and repair damaged cells and tissues (24, 25). Understanding the role of Tregs, as key inflammatory suppressive lymphocytes, in post-MI ventricular remodeling is helpful to develop effective therapeutic targets (26).

## 3 Characteristics and immunoregulatory roles of Tregs

Tregs are a subset of CD4^+^ T cells, accounting for 5%-10% of circulating CD4^+^ T cells. The features of Tregs include high expression of CD25 (IL-2 receptor alpha chain), high expression of transcription factor Foxp3, and low expression of CD127 (IL-7 receptor) (27). Foxp3 is the core molecule responsible for the function of Tregs, and its expression level directly determines the immunosuppressive capacity (28). Foxp3 deficiency leads to severe systemic inflammatory diseases (29). Tregs can be categorized into two types: natural Tregs derived from the thymus and induced Tregs generated from peripheral effector T cells (**Table 1**). They play different immunoregulatory roles under various physiological and pathological conditions (30).

**Table 1:** Characterization of Treg subsets.

	Natural Treg	Induced Treg
Source	Mature in the thymus	Induced by TGF-β, IL-2 in the periphery
Specific marker	CD4^+^ CD25^+^ CD127^−^ Foxp3^+^ Helios^+^	CD4^+^ CD25^+^ CD127^−^ Foxp3^+^
Stability	Stabilized express Foxp3	Unstable, may lose Foxp3 expression
Cytokine production	IL-10, TGF-β, IL-35	IL-10, TGF-β, IL-35
T cell receptor	Mainly recognize self-antigen	Mainly recognize foreign antigen
Main function	Suppress autoimmune disease and maintain immune tolerance	Play an important role in tumor, infection, transplant rejection and other pathological conditions

**Figure 1:**
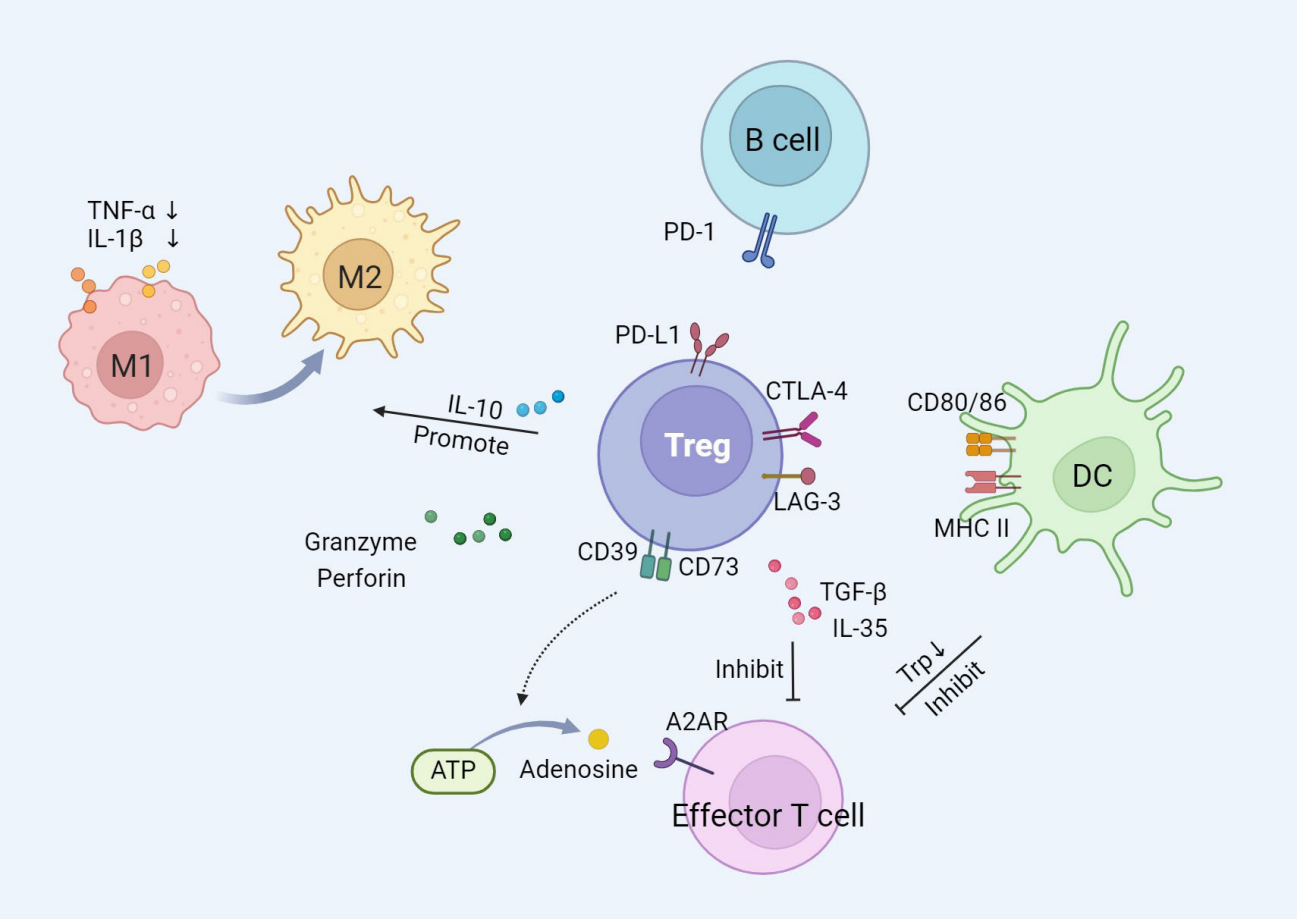
The immunosuppression mechanism of Tregs.

The immunomodulatory function of Tregs is primarily characterized by the suppression of over-activated immune response. Tregs exert immunosuppressive function on effector cells of innate and adaptive immunity, such as macrophages, neutrophils, dendritic cells (DCs), and T cells, through the utilization of multiple mechanisms (31). There are three main mechanisms by which Tregs exert their suppressive effects, including cell-to-cell interactions, cytokine release, and interference with target cell metabolism (**Figure 1**).

Tregs communicate directly with effector T cells or other immune cells through intercellular contact mechanisms. The surface of Tregs exhibits high expression of co-inhibitory receptors, including cytotoxic T-lymphocyte-associated protein 4 (CTLA-4), lymphocyte activation gene 3 (LAG-3), and programmed death-ligand 1 (PD-L1) (32). CTLA-4 competitively binds to B7 molecules (CD80, CD86) on the surface of DCs, preventing the interaction between CD28 and B7, thereby delivering inhibitory signals and suppressing the activation and proliferation of T cells (33). The binding of CTLA-4 to B7 mediates the expression of indoleamine 2,3-dioxygenase by DCs, which degrades tryptophan essential for T cell proliferation (34, 35). LAG-3 binds to MHC class II molecules on the cell membrane of DCs and inhibits the maturation of DCs through cytoplasmic signaling. PD-L1 binds to programmed death receptor 1 (PD-1) on B cells, inhibiting their proliferation and activation, and inducing B cell apoptosis (36).

Tregs release a variety of inhibitory cytokines, including interleukin (IL)-10, transforming growth factor-β (TGF-β), and IL-35, to regulate the immune response of target cells (37). IL-10 inhibits the secretion of two pro-inflammatory cytokines, tumor necrosis factor-α (TNF-α) and IL-1β, by monocytes and macrophages (38). It also induces the transformation of pro-inflammatory M1 macrophages into anti-inflammatory and pro-repair M2 macrophages (39). Anti-IL-10 neutralizing antibodies block the inhibition of effector T cells mediated by Tregs (40). Intracellular TGF-β induces an increase in Foxp3 expression, promoting the generation of Tregs (41). Blocking TGF-β signaling reduces Tregs-mediated immunosuppression and exerts anti-tumor therapeutic effects (42). Similar to TGF-β and IL-10, IL-35 plays an important role in immunomodulation and immune homeostasis by regulating the development of Tregs (43).

Tregs exert their suppressive function by interfering with the metabolic pathways of target cells. Specifically, Tregs are able to deliver a large amount of cyclic adenosine monophosphate (cAMP) to effector T cells, interfering with their glycolysis and oxidative phosphorylation processes, thereby reducing the energy supply and proliferative capacity of effector T cells (44). Furthermore, Tregs compete with effector T cells to consume IL-2, which is a crucial cytokine necessary for the proliferation and survival of effector T cells. By limiting the availability of IL-2, Tregs induce effector T cell death and immunosuppression (45, 46). Tregs express the exonucleases CD39 and CD73 on their surface, which catalyze the production of adenosine from ATP. The adenosine activates adenosine receptor A2 on effector T cells, exerting an inhibitory effect (37). In addition, Tregs can inhibit effector T cell-mediated tumor clearance through cytolysis. Tregs induce apoptosis in effector T cells by producing granzyme and perforin, thereby reducing the number of effector cells and mediating immune suppression (47).

## 4 Tregs improve ventricular remodeling after MI

Tang et al. amplified Tregs in MI rats by using adoptive transfer and CD28 superagonist antibody. They confirmed that increasing Tregs number in infarcted hearts prevented adverse ventricular remodeling and improved cardiac function (48). This study demonstrates for the first time that Tregs have an important regulatory role in post-MI remodeling. Lin et al. provided evidence that the level of Tregs in peripheral blood is significantly reduced in patients with ACS, compared to those with stable angina pectoris and individuals with normal coronary arteries (49). The migration of circulating Tregs to the site of cardiac inflammation leads to a decrease in the number of peripheral Tregs and an increase in the peri-infarct area. Furthermore, the frequency of Tregs is positively correlated with LVEF in ACS patients. Prospective studies on the relationship between Tregs and cardiovascular risk showed that low level of circulating Tregs were associated with a higher risk of MI (50).

A recent study showed that the number of circulating Tregs in patients with acute MI (AMI) at 72h was higher than that in healthy controls and patients with cardiovascular risks such as type 2 diabetes, dyslipidemia, and smoking. And the IL-10 secreted by Tregs was increased in AMI (7). The reason for this phenomenon may be that Tregs counteracted the inflammatory response following acute ischemia. Further analysis revealed that the percentage of Tregs in AMI patients remained relatively unchanged at 3 months but significantly decreased after 6 months. At the 3-month point after AMI, patients with LVEF greater than 50% have higher levels of Tregs. These studies suggest that the cardiac function seems to be influenced by the number of Tregs. Tregs may play a pivotal role as a potential therapeutic target in the progression and prognosis of MI.

## 5 The regulatory mechanism of Tregs in post-MI ventricular remodeling

### 5.1 Suppression of excessive inflammation

The inflammatory response triggered by AMI is an important factor in myocardial injury (9). Tregs limit the inflammatory response by inhibiting the infiltration of inflammatory cells at the site of myocardial injury. Within hours after AMI, there is an increase in the number of neutrophils in the infarct area, followed by monocytes/macrophages infiltration. This triggers an excessive inflammatory response (51). Expanding Tregs in the infarcted heart can reduce the infiltration of neutrophils, macrophages, and T lymphocytes, reduce the production of pro-inflammatory cytokines, inhibit the cytotoxic effects of CD8^+^ T lymphocytes, and mitigate ventricular remodeling (48). In contrast, Tregs depletion leads to an increased density of CD45^+^ inflammatory cells, significantly exacerbating both dilated and hypertrophic remodeling (52, 53). Reperfusion injury is a key pathophysiological process of revascularization in AMI. Selective depletion of Tregs exacerbated myocardial ischemia/reperfusion injury (MIRI), while injection of Tregs activated *in vitro* inhibited MIRI and alleviated ventricular remodeling (54). Studies on their mechanisms have shown that Tregs exert cardioprotective effects in a CD39-dependent manner by activating the AKT/ERK pathway, inhibiting cardiomyocyte apoptosis, and reducing neutrophil infiltration. When CD39 is deficient, this protective effect disappears, and the ability of Tregs to inhibit neutrophils is impaired. The IL-2/anti-IL-2 complex (IL-2C) can significantly expand Tregs in the spleen and heart of mice. Helper T cells (Th) 1 and Th17 secrete interferon-γ and IL-17 respectively, exacerbating the immune-inflammatory response after MI. IL-2C pretreatment before MIRI reduced the number of Th1 and Th17, decreased inflammatory cell infiltration after MIRI, and improved cardiac function. Moreover, IL-2C decreased the expression of pro-inflammatory cytokines TNF-α, interferon-γ, IL-12, and IL-17A in the heart (55).

Tregs improve adverse post-MI remodeling by modulating the ratio of pro-inflammatory/anti-inflammatory macrophages. Tregs promote the polarization of macrophages towards the M2 phenotype, which has anti-inflammatory and tissue repair properties, while inhibiting the M1-type polarization of pro-inflammatory macrophages, thereby facilitating the repair of damaged myocardial tissue (56). The research by Saha et al. supported this conclusion. They used intravenous infusion of neonatal mesenchymal stromal cells to enhance the proliferation of Tregs mediated by CD44, which promoted the differentiation of monocytes into M2 macrophage and improved the cardiac structure and function in MI rats (57). Zhang et al. also showed that exosomes secreted by DCs activated Tregs to transform macrophages into M2 type. This resulted in a significant increase of Tregs and M2 macrophages infiltration in the border zone of AMI mice, thereby improving cardiac function (58). IL-2C inhibited the expression of pro-inflammatory cytokine genes and macrophage infiltration in the MI region, and induced the differentiation of macrophages from M1 to M2 phenotype in the border zone. IL-2C therapy may be a potential way to improve ischemic heart disease (59). All of these results suggest that Tregs promote macrophage differentiation toward an anti-inflammatory phenotype. In a mouse model of Tregs depleted by anti-CD25 antibody, the proportion of pro-inflammatory macrophages in the MI region was increased (56). Tregs inhibited the activation of pro-inflammatory macrophages. In MIRI mice, CXC chemokine receptor 4 antagonists reduced the expression of inflammatory genes in monocytes and macrophages by mobilizing splenic Tregs to the infarct zone (60).

Jia et al. investigated the mechanism by which Tregs promote the survival of anti-inflammatory macrophages. IL-35 is mainly expressed in Tregs. Ly6C^low^ is a monocyte/macrophage subtype with anti-inflammatory effects. IL-35 promoted the survival of Ly6C^low^ macrophages by activating the expression of CX3CR1 and TGF-β1 in macrophages through the phosphorylation of STAT1 and STAT4. IL-35 inhibition reduced the survival of Ly6C^low^ macrophages in infarcted heart. This resulted in impaired healing of the infarct area and aggravated cardiac remodeling (61). Feng et al. showed that chemokine ligand 17 (CCL17) was expressed in CCR2^+^ macrophages and DCs infiltrating the heart after myocardial injury. CCL17 inhibited Tregs recruitment through activation of CCR4 and exacerbated ventricular remodeling in MIRI mice. The absence of CCL17 led to an increase in the number of Tregs in the myocardium, reduced left ventricular remodeling, and improved systolic function. Increased Tregs further inhibited the expression of pro-inflammatory cytokines and chemokines in macrophages (6). This study reveals a potential link between Tregs and macrophages in post-MI ventricular remodeling. The interaction between Tregs and macrophages may be conducive to the formation of an anti-inflammatory microenvironment. Additionally, Tregs may influence macrophage polarization through the release of exosomes. Hu et al. found that exosomes secreted by Tregs after MI promoted anti-inflammatory macrophage polarization, reduced infarct size and inhibited cardiomyocyte apoptosis (62).

In summary, Tregs improve post-MI ventricular remodeling by inhibiting the infiltration of inflammatory cells, regulating macrophage differentiation, and reducing the expression of inflammatory cytokines.

### 5.2 Inhibition of cardiomyocyte apoptosis

Tregs play a beneficial role in adverse post-MI ventricular remodeling by modulating cardiomyocyte proliferation and apoptosis. In a lipopolysaccharide-induced inflammation model *in vitro*, Tregs reduced apoptosis of neonatal rat cardiomyocytes through direct intercellular contact and the secretion of IL-10 (48). Another study found that Tregs increased the phosphorylation levels of Akt and ERK1/2 in cardiomyocytes of MIRI mice. This confirmed that Tregs inhibited cardiomyocyte apoptosis by activating the ERK/Akt signaling pathway (54). Furthermore, Tregs can promote cardiomyocyte proliferation. Li et al. performed single-cell RNA sequencing on Tregs. They found that Tregs could promote neonatal cardiomyocyte proliferation by paracrine secretion of factors such as CCL24, GAS6, or AREG (63). Zacchigna et al. detected proliferative cardiomyocytes in the hearts of pregnant mice, and Tregs depletion during pregnancy reduced the proliferation of maternal and fetal cardiomyocytes. The researchers also injected Tregs into the area surrounding ischemia, which resulted in an increase in cardiomyocyte proliferation and a decrease in infarct size. Further studies have shown that Tregs promote cardiomyocyte proliferation and improve post-MI remodeling through paracrine secretion of cytokines such as Cst7 (53).

Apoptosis is closely related to inflammatory response. Anti-inflammatory effects help protect cardiomyocytes from attack by excessive immune response, which in turn reduce cardiomyocyte apoptosis. Recently, Wang et al. constructed a drug-loaded microgel system that responds to reactive oxygen species stimulation, effectively converting pro-inflammatory Th17 into anti-inflammatory Tregs. This system reduced early cardiomyocyte apoptosis post-MI, decreased inflammation *in vivo*, and protected cardiac function (64). The microgel system opens a new avenue for immunotherapy to improve ventricular remodeling.

### 5.3 Regulation of cardiac fibroblast function

Tregs participate in regulating fibroblast phenotype, inhibiting myocardial fibrosis and ventricular remodeling, and play a positive role in recovery after MI. Ramjee et al. observed that loss of IFN-γ in the myocardium led to reduced Tregs recruitment and enhanced fibrotic response in damaged myocardium post-MI (65). The number of Tregs in the left ventricular free wall of mice treated with IFN-γ doubled, and the area of myocardial fibrosis was relatively reduced by 34.4%. It suggested that IFN-γ recruited Tregs to the infarct area, limiting excessive myocardial fibrosis. *In vitro* co-culture experiment of Tregs with cardiac fibroblasts showed that Tregs regulated the function of cardiac fibroblasts. Tregs decreased the expression of α-smooth muscle actin and MMP-3, and attenuated collagen contraction (52).

However, Tregs may have a dual role in regulating myocardial fibrosis. Tregs facilitate the formation of new extracellular matrix and promote myocardial fibrosis after AMI by enhancing collagen deposition. After MI, Tregs were activated with superagonistic CD28-specific monoclonal antibody, which increased the level of TGF-β1 in myocardium (56). TGF-β1 promoted collagen synthesis in myofibroblasts. Increased collagen content and maturation in the infarct area of mice resulted in the formation of stable scar tissue, which prevented post-infarction left ventricular dilatation and rupture, as well as undesirable remodeling of the surviving myocardium.

Although the role of Tregs in cardiac fibrosis remains controversial, the above findings confirm the importance of Tregs in ventricular remodeling. Future studies may provide insight into the complex balance between the pro-fibrotic and anti-fibrotic effects of Tregs. It could help to develop new strategies for the treatment of MI-related ventricular remodeling.

### 5.4 Promoting angiogenesis

Angiogenesis is a critical process in heart repair, helping to restore blood supply to ischemic areas, reduce cardiomyocyte death, and promote recovery of cardiac function. Administering exogenous Tregs to MI mice resulted in a significant increase in the number of small capillaries within the infarcted heart, as demonstrated by CD31 staining (66). While ablation of Tregs led to a decrease in the number of small capillaries. Xiao et al. also observed an increase in small vessel density following Tregs amplification in the MIRI model (55). These results indicate that Tregs play an important role in microangiogenesis.

Tregs may promote angiogenesis by affecting macrophage polarization. Tregs are able to regulate monocytes/macrophages from a pro-inflammatory to a pro-repair state. This transformation involves an increase in the secretion of angiogenic factors by macrophages, such as vascular endothelial growth factor and fibroblast growth factor, which contribute to angiogenesis (67, 68).

IL-10 secreted by Tregs is a mediator that induces angiogenesis after MI. On the one hand, IL-10 can inhibit the production of pro-inflammatory cytokines, reducing their inhibitory effects on angiogenesis. On the other hand, IL-10 can also directly act on vascular endothelial cells, promoting their proliferation and migration, thus accelerating the formation of new blood vessels (30). Tregs can also promote endothelial cell proliferation by increasing the expression of Apelin, a cardiovascular active peptide (69). It follows that Tregs may promote angiogenesis through multiple mechanisms.

## 6 Clinical application prospects of Tregs

Although there is plenty of evidence supporting the benefits of Tregs in animal models of MI, it often takes a long time to translate these benefits into practical clinical applications for patients. Conventional tools for Tregs amplification include antibodies such as anti-CD3/CD28, and cytokines such as IL-2. As early as 2006, research institutions attempted to develop immunotherapies that activate endogenous Tregs. They conducted a Phase 1 trial using Tregs agonists in six healthy young volunteers (70). Within 90 minutes after receiving intravenous injection of the super-agonist anti-CD28 monoclonal antibody (TGN1412), all subjects experienced systemic inflammatory reactions, including headache, myalgia, nausea, diarrhea, and hypotension. Twelve hours later, patients were in critical condition and received cardiopulmonary support in the intensive care unit. Although all six subjects ultimately survived, this case nevertheless reveals the complexity and high risk of Tregs-activated therapy. A study conducted by Tian et al. evaluated the safety and efficacy of low-dose IL-2 in patients with ischemic heart disease (71). They demonstrated that low-dose subcutaneous injections of IL-2 successfully elevated Tregs levels without significant adverse effects. This indicates that low-dose IL-2 may have potential to treat ischemic heart disease. In addition to the activation of endogenous Tregs, the adoptive transfer of exogenous Tregs has also shown significant promise in improving post-MI ventricular remodeling (46, 48). It can be divided into autologous Tregs reinfusion and allogeneic Tregs transplantation. Autologous Tregs reinfusion has a lower risk of immune rejection. However, its preparation process is time-consuming and is influenced by the quantity and functionality of the patient’s own Tregs, which limits its application in AMI. In contrast, off-the-shelf allogeneic Tregs derived from healthy donors can be prepared and stored in advance, making them suitable for rapid treatment. Nevertheless, allogeneic Tregs may trigger host immune rejection, and their long-term survival and functional stability still require further optimization (26). Therefore, autologous Tregs may be more suitable for the regulation of chronic inflammation, while allogeneic Tregs may be more appropriate for rapid intervention during the acute phase of MI. In the future, large-scale studies will be needed to confirm its safety and efficacy.

Tregs play a crucial role in maintaining immune homeostasis through their antigen specificity. Antigen-specific Tregs suppress immune responses against specific antigens, demonstrating significant therapeutic potential in autoimmune diseases and transplant tolerance (72). However, the pathological process of MI involves multiple antigens and extensive inflammatory responses. Polyclonal Tregs possess a broader antigen recognition capability, enabling them to suppress multiple immune responses simultaneously, which may confer an advantage in the treatment of MI. Nonetheless, their non-specific suppression may increase the risk of infections or tumorigenesis. Thus, careful consideration of the balance between efficacy and safety remains essential for their clinical application.

In recent years, the clinical application of chimeric antigen receptor T (CAR-T) cell therapy is rapidly developing. CAR is a modified receptor that directs T cells to recognize and eliminate cells expressing the target antigen. Excessive myocardial fibrosis is known to be a cause of adverse ventricular remodeling after AMI. CD5-targeted lipid nanoparticles encapsulating modified mRNA can transiently generate CAR-T cells targeting fibroblast activation protein, reduce myocardial fibrosis and improve cardiac systolic and diastolic function in mouse models of heart failure (73). Targeted regulation of fibroblast activation protein provides new research insights for CAR-Treg cells to improve ventricular remodeling after MI.

Heart transplantation is an effective treatment for patients with end-stage heart failure, but it is limited by allograft rejection. Increasing the number of Tregs or enhancing their suppressive function can help prevent allograft rejection. In a mouse model of allogeneic heart transplant rejection, low-dose IL-2 increased Tregs infiltration in the spleen and graft, prolonged graft survival, and prevented chronic rejection (74). Studies have shown that the combination of matrine and tacrolimus can alleviate acute rejection in mouse allogeneic heart transplantation by inhibiting the maturation of DCs and increasing the proportion of Tregs, thereby mediating immune tolerance post-heart transplantation (75).

Adoptive transfer of exogenous Tregs or amplification of endogenous Tregs has demonstrated protective effects on post-MI ventricular remodeling. Extensive basic experimental research and rigorous evaluations of efficacy and safety are essential before conducting clinical studies. Future research should also address how to optimize the Tregs amplification pathway, how to maintain their stability, and how to direct them to target the infarct area, in order to successfully translate this new strategy into an effective clinical approach for the treatment of MI.

## 7 Conclusions

**Figure 2:**
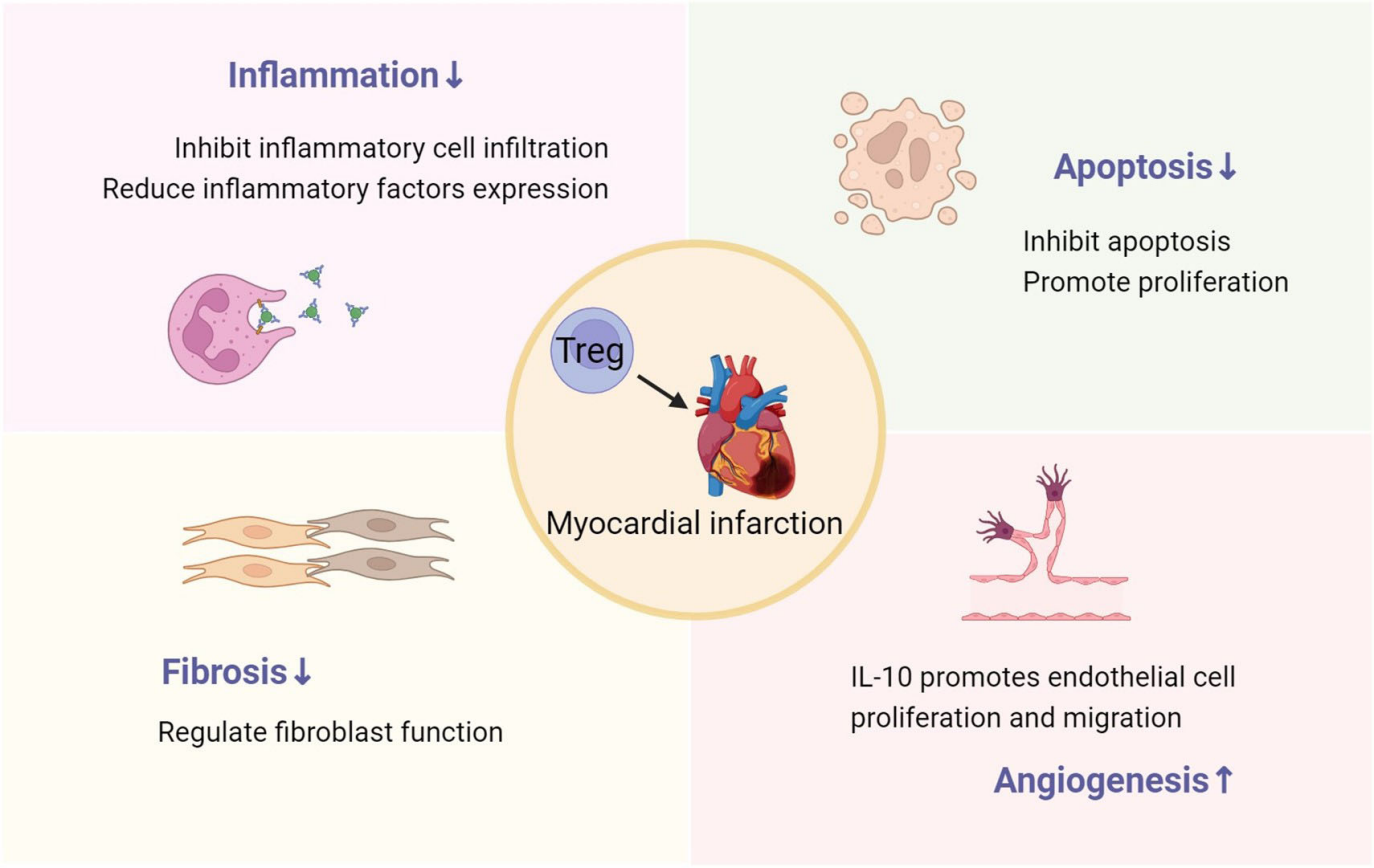
The role of Tregs in post-MI ventricular remodeling, including inhibiting inflammation, reducing cardiomyocyte apoptosis and fibrosis, and promoting angiogenesis.

Increasing the function or number of Tregs can ameliorate post-MI ventricular remodeling, with the primary mechanisms encompassing the suppression of inflammatory response, reduction of cardiomyocyte apoptosis, modulation of cardiac fibroblast function, and promotion of angiogenesis (**Figure 2**). Tregs are considered as potential therapeutic targets. In recent years, a growing number of studies have focused on facilitating the transition of Tregs from basic research to clinical applications. The development of biomimetic nanoparticles and engineered Tregs has also brought promises in this therapeutic field (76, 77). Future research needs to further delve into the role and mechanisms of Tregs after MI, and carry out necessary clinical trials. Effective utilization of Tregs may provide a new potential avenue for the treatment of MI.
